# Thermal face recognition under different conditions

**DOI:** 10.1186/s12859-021-04228-y

**Published:** 2021-11-08

**Authors:** Shinfeng D. Lin, Luming Chen, Wensheng Chen

**Affiliations:** Department of Computer Science and Information Engineering, National Dong Hwa University, Hualien, Taiwan

**Keywords:** Convolutional neural network, Face recognition, Thermal image, Bayesian framework, Random forest

## Abstract

**Background:**

A thermal face recognition under different conditions is proposed in this article. The novelty of the proposed method is applying temperature information in the recognition of thermal face. The physiological information is obtained from the face using a thermal camera, and a machine learning classifier is utilized for thermal face recognition. The steps of preprocessing, feature extraction and classification are incorporated in training phase. First of all, by using Bayesian framework, the human face can be extracted from thermal face image. Several thermal points are selected as a feature vector. These points are utilized to train Random Forest (RF). Random Forest is a supervised learning algorithm. It is an ensemble of decision trees. Namely, RF merges multiple decision trees together to obtain a more accurate classification. Feature vectors from the testing image are fed into the classifier for face recognition.

**Results:**

Experiments were conducted under different conditions, including normal, adding noise, wearing glasses, face mask, and glasses with mask. To compare the performance with the convolutional neural network-based technique, experimental results of the proposed method demonstrate its robustness against different challenges.

**Conclusions:**

Comparisons with other techniques demonstrate that the proposed method is robust under less feature points, which is around one twenty-eighth to one sixtieth of those by other classic methods.

## Background

Face recognition is critical in many applications, including criminal identification, video surveillance, and smart city. It can be performed by using different feature extraction and classification techniques. Because of the low cost and availability of conventional CCD/CMOS cameras, most of the existing approaches have been biased towards the visible spectrum for face recognition. Unfortunately, it is still a challenge to achieve robust face recognition in real-world environment [1, 2]. There are some variations causing the trouble of face recognition in visible spectrum such as pose, illumination, and disguises. The face recognition in visible spectrum is still one of the most active research topic.

To solve the problem of illumination changes in visible spectrum, one solution is to use a 3D device that is not so sensitive to lighting variations. However, the processing speed of the system is not so efficient. The problem above may be overcome by the techniques of infrared image. The infrared (IR) spectrum is usually divided into four sub-bands: (1) wavelength 0.75–1.4 μm—Near IR (NIR), (2) wavelength 1.4–3 μm—Short Wave IR (SWIR), (3) wavelength 3–8 μm—Medium Wave IR (MWIR), (4) wavelength 8–15 μm—Long Wave IR (LWIR). The thermal infrared imagery of the wavelength ranges in the 0.35 to 0.74 μm [3–5]. Thermal radiation has several benefits compared to visible light. Moreover, thermal infrared can be transmitted in bad illumination environment including complete darkness.

Nowadays, researchers have investigated the techniques and applications of thermal infrared imagery such as lie detection [6], and human activity recognition [7]. Thermal cameras can detect thermal radiation emitted from an object, convert this radiation to temperature, and display an image of temperature distribution [8–10]. As we know, people undergoes physiological changes while facing stress. Zhu et al. [6] proposed a segmentation for the extraction of forehead signatures in thermal video clips which can further be used in deception detection. It depends on tracking a forehead Region of Interest (ROI). By using robust features and a deep recurrent neural network, Uddin and Torresen [7] proposed a thermal camera-based human activity recognition. The proposed approach is very useful to monitor humans in dark environments which is superior to the RGB cameras.

It is attractive to consider thermal sensors in face recognition, due to the development of thermal infrared technology. Skin temperature can be visualized and measured with a thermal camera. Generally, human facial skin temperature is closely related to the underlying blood vessels. Many factors (physiological, environmental, and imaging conditions) may affect the thermal imaging of a human face [11, 12]. A facial thermal pattern, which is unique, is decided by the vascular structure of each face. While taking the images at different times, there is little change on its structure [13, 14]. These constant thermal features will be utilized to match the thermal signature to a specific individual. A technique analogous to fingerprint recognition [15] is adopted for identifying facial identities.

Buddharaju et al. [16] presented a recognition system based on characteristic and time-invariant physiological information. The superficial blood vessel network was localized with image processing technology. By using white top hat segmentation, the vascular structure was acquired from the surface of the skin. Then, Thermal Minuta Points (TMP)-based feature vectors were employed for recognition. Vigneau et al. [17] analyzed the problems resulting from temporal variations of infrared face images. They used five traditional feature-based methods to develop a thermal face recognition. Hermosilla et al. [18] proposed a computer vision system based on the DrunkSpace. The dimensionality of the feature vectors was reduced with Fisher linear discriminant (FLD) method to construct a subspace called DrunkSpace. A Bayesian classifier based on Gaussian mixture models (GMM) is exploited to identify if an individual is drunk.

We propose a thermal face recognition under different conditions, motivated by [18], in this paper. The most representative points on the face are chosen for references [19]. The positions of several points are based on the veins and capillaries that cross the face. These points are selected as a feature vector, and the Random Forest algorithm [20] is adopted to construct the classifier. The RF algorithm is a supervised learning algorithm that can merge multiple decision trees together to obtain a more accurate classification result. During the testing phase, the corresponding feature vectors are extracted from testing images and inputted into the classifier for the identity of the individual. For performance evaluation of the proposed method, experiments will be conducted under different conditions, including normal, adding noise, wearing glasses, and face mask. This system is able to recognize the identity of an individual using the information from the thermal image. The novelty of our method is to adopt the most representative thermal information on the face as a feature vector for classifier training. In comparison with the performance of the CNN-based technique [21], experimental results of the proposed method demonstrate its feasibility against different challenges.

## Methods

**Fig. 1:**
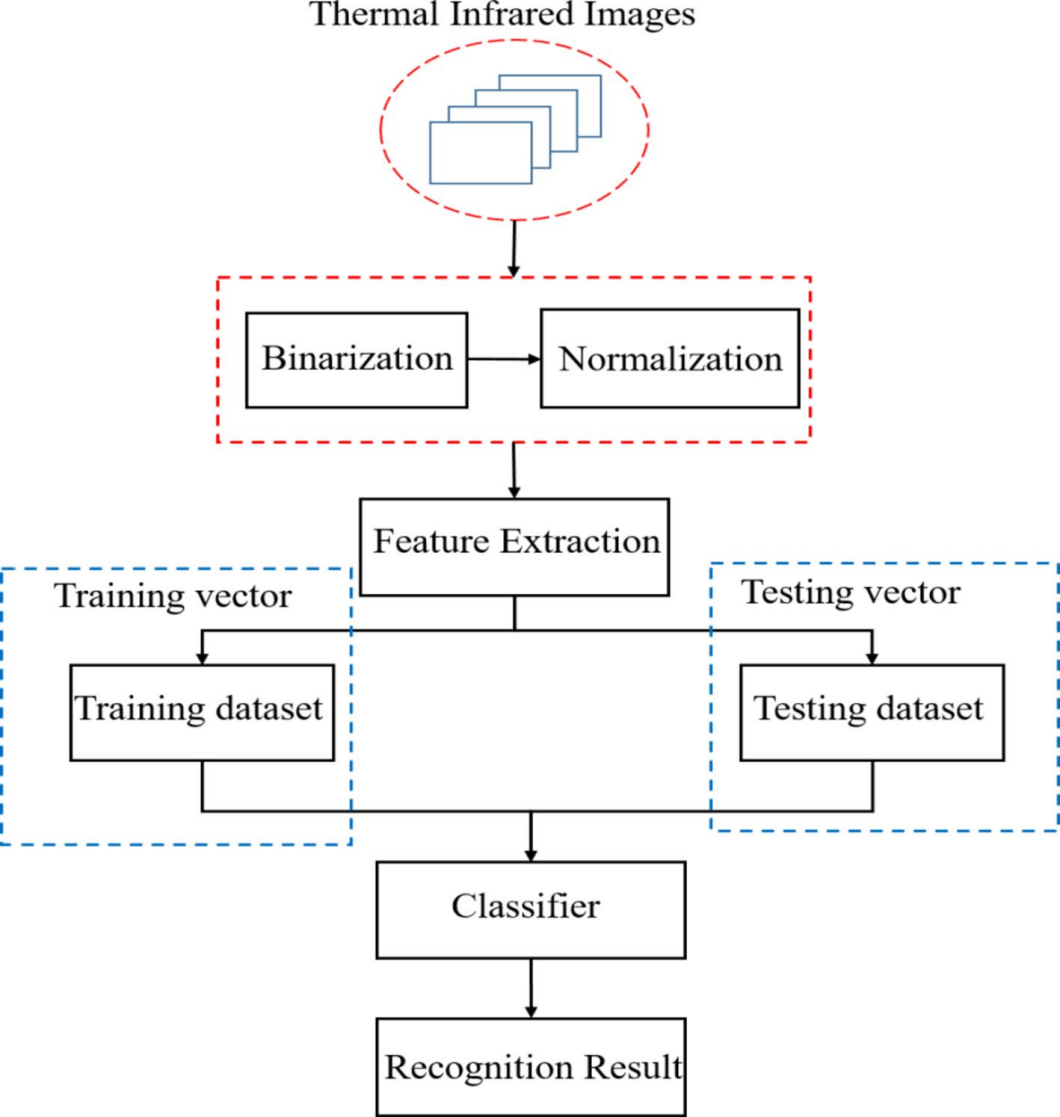
The proposed flowchart of thermal face recognition

As can be seen in Fig. 1, the proposed flowchart of thermal face recognition has two phases: training and testing phases. During the training phase, it contains three steps: (1) preprocessing, (2) feature extraction, (3) classification. First, the human face is acquired with the Bayesian framework [16] from the thermal image, and the face image is then normalized to a uniform size. A grid of several points is extracted from each of the thermal images to generate a feature vector. This vector is then used for training RF classifier. During the testing phase, the corresponding feature vectors are extracted from the testing images and inputted into the classifier for face identification.

### Dataset

For the evaluation of the proposed method, the PUCV Drunk Thermal Face (PUCV-DTF) [18] and the UCH Thermal Temporal Face (UCH-TTF) [17] databases are used to conduct experiments. While a great number of databases designed for different tasks, only some relevant thermal face databases have been presented so far. To further verify the feasibility of the proposed method in a real environment, we extend the scope of the experiment. The original images are modified in both databases for simulating real conditions such as noise, and occlusion. A content description of each database is addressed as follows.

**Fig. 2:**
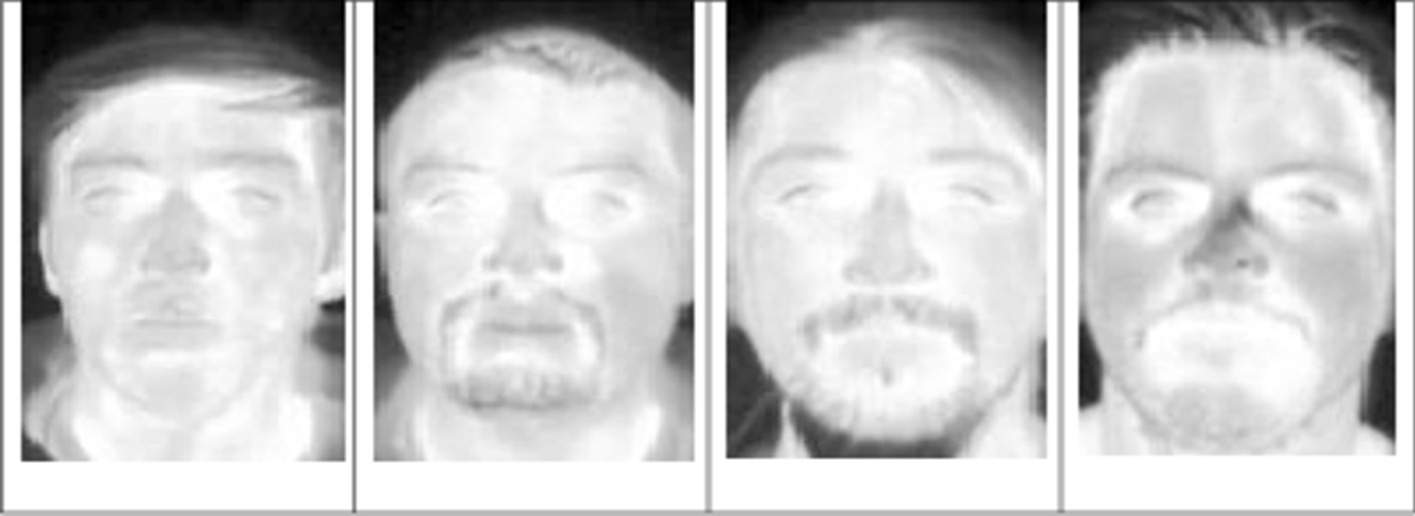
The sample images in PUCV-DTF database

The samples of the PUCV-DTF database is shown in Fig. 2. These images are taken over time using FLIR Tau2 thermal imaging cameras [22]. This thermal database includes 46 people, each of them has five subsets, and each subset has 50 images. This results in a total of 250 images. In preprocessing, according to the coordinates of the eyes, each image is cropped and aligned. This leads to a resolution of 81 × 150 pixels.

**Fig. 3:**
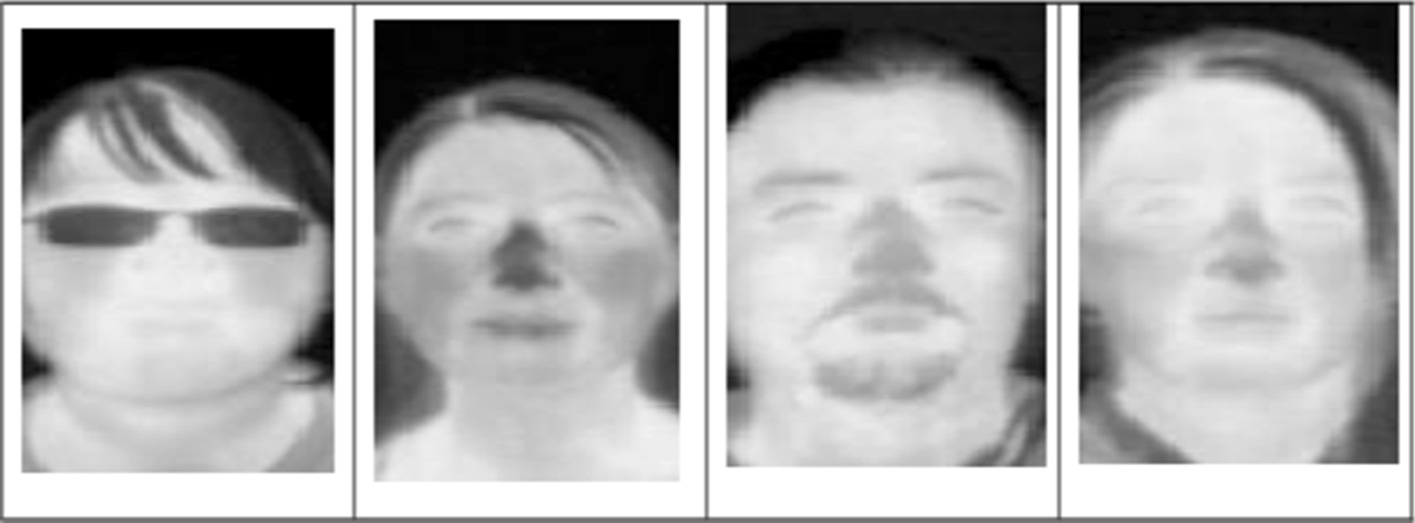
The sample images in UCH-TTF database

Figure 3 shows the samples of the UCH-TTF database. These images are taken from 7 different people, each of them has 50 images, by using a FLIR TAU 320 thermal cameras [23]. In preprocessing, the images are cropped and aligned to 150 * 81 and 125 * 225 pixels.

**Fig. 4:**
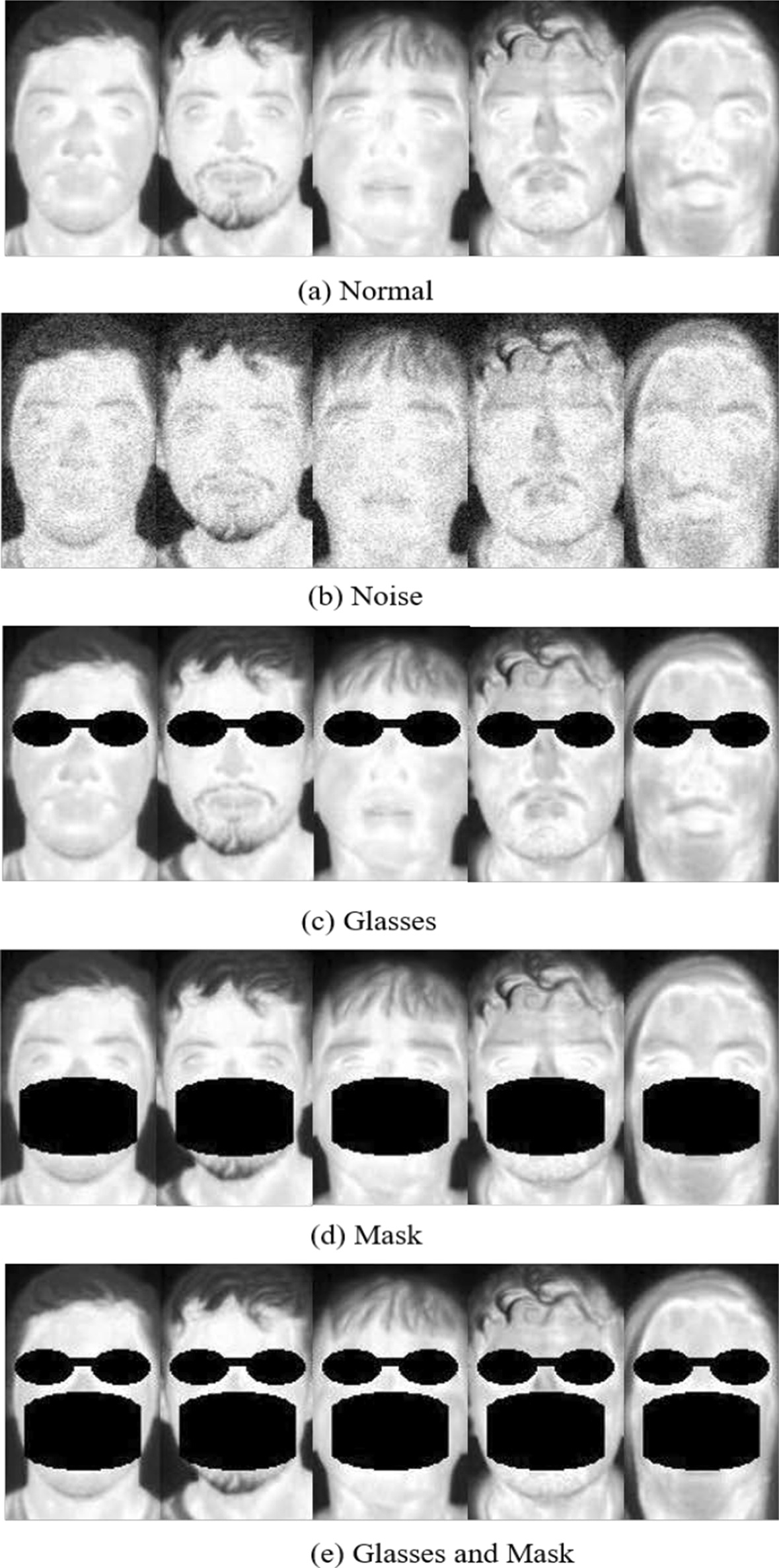
The original and modified experiment samples **a** Normal images **b** Noise images **c** Glasses images **d** Mask images **e** Glasses and Mask images

Figure 4 shows five different experiment samples, including normal (original images), noise (with noise), glasses (in glasses), mask (in mask), and both (in glasses and mask). The normal image is shown in Fig. 4a. As the most common noise in thermal images [24], Gaussian noise is applied on thermal images, as shown in Fig. 4b. Glasses are opaque to most of the thermal spectrum, including LWIR, MWIR, and SWIR [5]. This means that a portion of the face might be occluded when wearing glasses, causing the loss of information near the eyes. In Fig. 4c, a specific mask in the eye position is added to the original images for simulating the wearing of glasses. To simulate face mask, a little bit nose and the mouth of the original images are masked, as shown in Fig. 4d. In Fig. 4e, to simulate the wearing of glasses and mask, both specific masks are added to the original images.

### Feature extraction

**Fig. 5:**
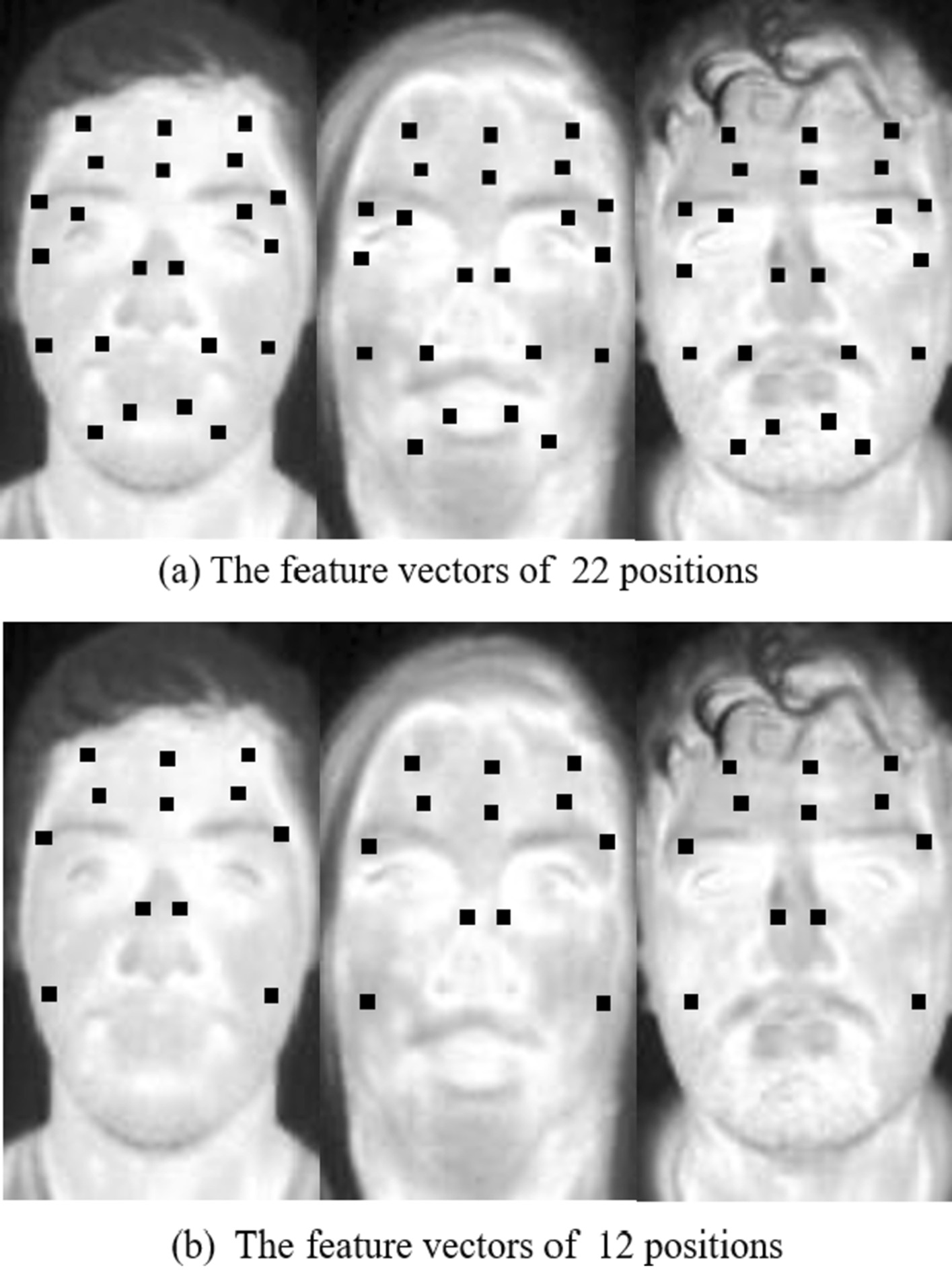
Feature vectors **a** 22 positions **b** 12 positions

The most representative thermal points on the face are selected as a feature vector and used for the classifier training. In Fig. 5, each black block is a neighborhood of 3 × 3 pixels. The average intensity of every black block is computed to compensate for the influence of noise. In [18], a different grid of 22 points was chosen from different regions of the face, as shown in Fig. 5a. Figure 5b shows the feature vectors of 12 positions, which might not be occluded by glasses and mask.

### Random forest classification

RF [20] can be used for solving different classification problems. The main idea of the algorithm is based on multiple decision tree to construct an optimal classification model. This algorithm is also a variant of the bagging algorithm in the training process of decision trees. It can build a number of de-correlated trees to reduce the correlation between trees. Therefore, the performance of generalization is improved.

During the training phase, RF algorithm is employed to construct the classifier for multiclass classification. The feature vectors are used for classifier training. These vectors are fed to each decision tree, and each tree votes for a class. Finally, the class belonging of the object is decided according to the highest number of votes.

The implementation of the RF is based on Scikit-Learn package. Firstly, the RandomizedSearchCV method of Scikit-Learn was used to do the Hyperparameter Tuning of the Random Forest for getting the best parameter of RF. The parameters of RF is listed as {'criterion': 'gini', 'max_depth': 70, 'n_estimators': 700, 'n_jobs': -1, 'random_state': 777, 'warm_start': True}.

### Convolutional neural network

CNN [21] is a class of neural networks commonly used to analyze visual imagery in deep learning. It can be used to solve complex image classification problems [25, 26]. CNN consists of different layers that include the Input layer, Output layer, Convolution layer, Pooling layer, Flatten layer, and Fully connected layer (FC). The convolutional layers effectively process images to extract features from training sets. By the overlap of the small images, these layers maintain the spatial relationship among the pixels. FC layers calculate the predicted values of the test image through the feature vector of the last convolution layer. The recognition result is the category related to the highest probability. In this study, the architecture of CNN is designed referring to VGGNet [27]. VGGNet’s weight configuration has been used as a baseline feature extractor, it is publicly available. VGG16 architecture weights are quite large, which may be a little bit difficult to handle. The simple and better structure in CNN model is decided by choosing the best accuracy associated with different kernel configurations and architectures. Table 1 lists our architecture of CNN.

**Table 1:** Our architecture of CNN

Convolution layer-1	(5 * 5, 16)
maxpool	
Convolution layer-2	(3 * 3, 32)
maxpool	
Convolution layer-3	(3 * 3, 46)
maxpool	
Convolution layer-4	(3 * 3, 128)
maxpool	
FC-1024	
FC-1024	
soft-max	

## Results

In this section, the proposed method is realized in Python language. The experiments are conducted on Windows PC with 3.2 GHz and 8G RAM.

### Different feature vector sizes

In Table 2, parameter F represents the size of the feature vector, and parameter N indicates the images per individual used for the classifier training. The model is trained using the normal image, and tested using images with glasses and mask. For example, N = 30 means we adopt the first 30 images in database for training, and test the last 20 images out of 50 images with glasses and mask. The experiment is conducted to analyze the performance of different feature vector size under occlusion (in glasses and mask). This analysis will allow us to decide the size of feature vectors with the best performance in the case of occlusion. The results of the experiments are compared to conclude which of the four feature vector size gives the best performance. In both databases, the size of feature vectors for F = 12 outperforms the rest of sizes.

**Table 2:** Performance of images with glasses and mask (both databases)

Glasses and mask	Normal					
	N = 5	N = 10	N = 15	N = 20	N = 25	N = 30
PUCV-DTF						
10	97.57	99.13	99.96	100	100	100
12	98.96	100	100	100	100	100
14	96.63	98.46	98.59	99.35	99.57	99.63
22	35.17	36.65	36.57	37.04	37.91	38.65
UCH-TTF						
10	76.14	82.57	83.29	83.57	85.57	86.86
12	77.57	84.61	84.86	86.86	89.57	90.14
14	75.14	82.43	82.57	83.29	84.29	86.14
22	27.14	29.43	29.86	30.29	31.43	32.71

### Three different experiments

**Fig. 6:**
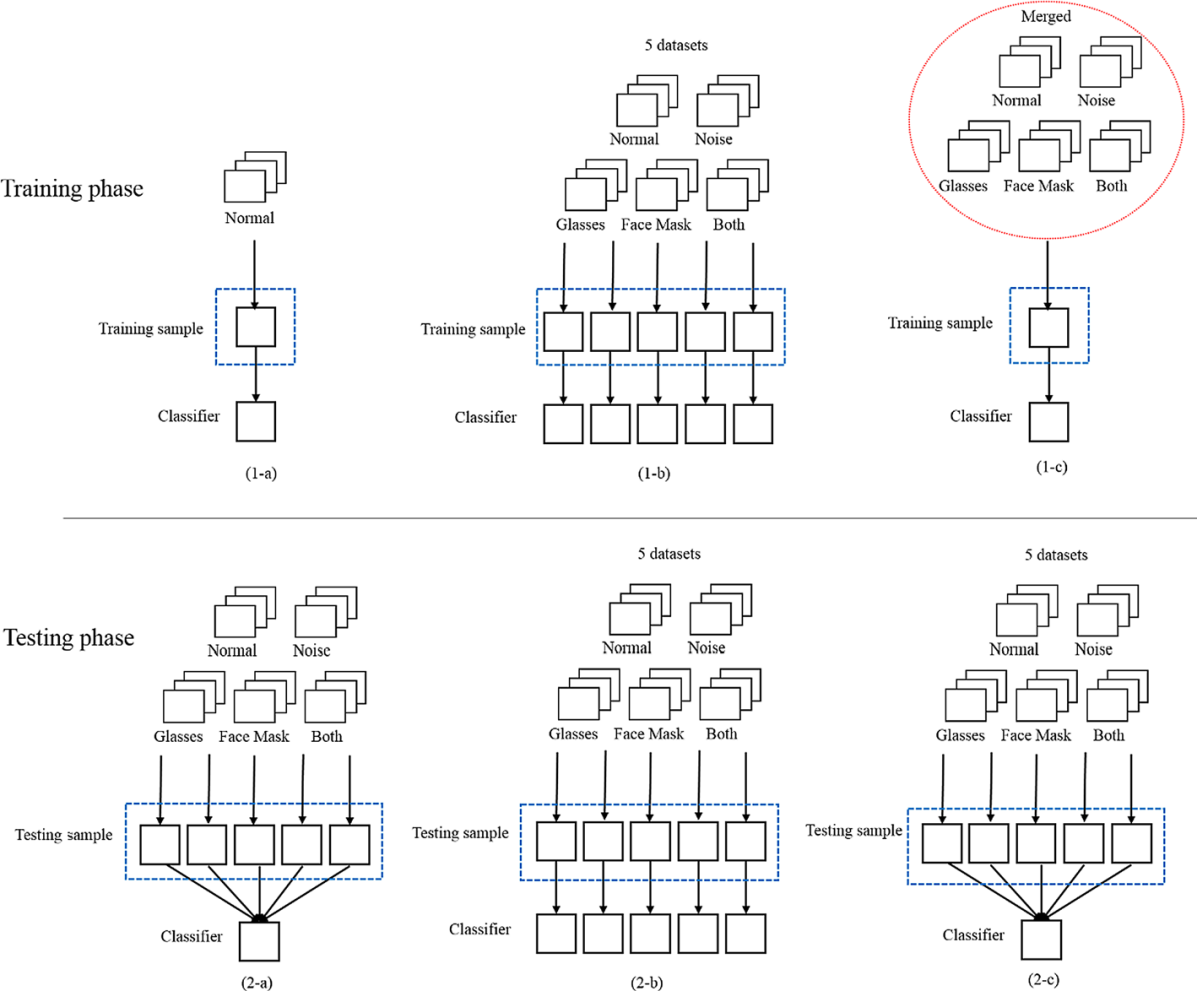
Three different experiments. **1-a**, **2-a** The normal image is trained and each image dataset is tested separately, **1-b**, **2-b** Each experiment image is utilized for training and testing, respectively, **1-c**, **2-c** All experiment images are merged for training set, and each experiment image is tested separately

Figure 6 presents three different experiments. Five experiment images, as shown in Fig. 4, are used to conduct these experiments. In the first experiment, the normal image is trained and each image dataset is tested separately, as shown in Fig. 6 1-a and 2-a. Each experiment image is utilized for training and testing, respectively, as shown in Fig. 6 1-b and 2-b. In Fig. 6 1-c and 2-c, all experiment images are merged for training set, and each experiment image is tested separately.

### Performance evaluation

Tables 3 and 4 present results of three different experiments, each of them has five experiment images, including normal, noise, glasses, mask, and both. The experiment results are obtained by RF approach with 22 and 12 thermal points. With normal and noise images of the PUCV-DTF database in Table 3, experimental results for F = 22 are very impressive, even the number of training images is not many. When the size of feature vectors is reduced, the performance will decrease, as shown in Table 4. It is obvious that the size of feature vectors determines the recognition rate. With the occluded images, the first experiment proves that the recognition rate of RF approach with 22 thermal points is not satisfactory.

**Table 3:** Performance of three different experiments for F = 22

F = 22	Normal (I)						5 datasets (II)						Merged (III)					
	N = 5	N = 10	N = 15	N = 20	N = 25	N = 30	N = 5	N = 10	N = 15	N = 20	N = 25	N = 30	N = 5	N = 10	N = 15	N = 20	N = 25	N = 30
PUCV-DTF																		
Normal	99.13	100	100	100	100	100	99.13	100	100	100	100	100	99.48	100	100	100	100	100
Noise	88.91	89.43	89.61	90.09	90.14	90.22	98.26	99.13	99.48	99.74	99.87	99.91	95.87	96.74	97.74	97.83	98.61	99.74
Glasses (G)	55.48	55.57	56.09	58.65	60.26	60.65	99.78	100	100	100	100	100	98.26	99.57	100	100	100	100
Face mask (FM)	47.61	48.14	48.57	49.13	50.83	51.35	99.35	100	100	100	100	100	97.13	98.04	98.57	100	100	100
G + M	35.17	36.65	36.57	37.04	37.91	38.65	99.57	100	100	100	100	100	95.26	97.35	98.57	99.04	99.91	100
UCH-TTF																		
Normal	85.71	87.14	90.71	92.14	92.86	95.71	85.71	87.14	90.71	92.14	92.86	95.71	83.29	84.43	85.71	87.43	89.29	92.29
Noise	73.86	75.71	77.43	79.71	81.86	83.14	83.57	84.29	85.71	86.43	87.86	89.29	79.29	79.29	81.43	83.14	86.14	88.0
Glasses (G)	45.43	45.86	46.14	47.71	48.14	48.86	84.14	86.86	87.86	88.29	89.71	90.71	78.71	83.43	83.57	84.29	86.43	87.14
Face mask (FM)	40.14	41.14	43.14	42.29	42.57	43.86	83.86	85.14	86.71	87.86	89.57	89.86	76.43	79.86	80.86	81.29	84.29	85.71
G + M	27.14	29.43	29.86	30.29	31.43	32.71	81.43	85.29	86.29	87.17	89.86	89.29	75.14	78.57	79.29	79.29	83.57	83.57

**Table 4:** Performance of three different experiments for F = 12

F = 12	Normal (I)						5 datasets (II)						Merged (III)					
	N = 5	N = 10	N = 15	N = 20	N = 25	N = 30	N = 5	N = 10	N = 15	N = 20	N = 25	N = 30	N = 5	N = 10	N = 15	N = 20	N = 25	N = 30
PUCV-DTF																		
Normal	98.96	100	100	100	100	100	98.96	100	100	100	100	100	98.57	100	100	100	100	100
Noise	86.2	86.74	87.2	87.61	88.28	88.83	97.39	98.37	98.48	99.02	99.35	99.61	95.96	96.13	97.22	97.57	98.09	98.87
Glasses (G)	98.96	100	100	100	100	100	98.96	100	100	100	100	100	98.57	100	100	100	100	100
Face mask (FM)	98.96	100	100	100	100	100	98.96	100	100	100	100	100	98.57	100	100	100	100	100
G + M	98.96	100	100	100	100	100	98.96	100	100	100	100	100	98.57	100	100	100	100	100
UCH-TTF																		
Normal	77.57	84.61	84.86	86.86	89.57	90.14	77.57	84.61	84.86	86.86	89.57	90.14	77.14	84.29	84.86	86.71	88.57	90.0
Noise	64.29	69.71	75.71	77.86	79.29	80.0	74.86	76.14	81.43	84.71	86.29	88.29	72.86	75.71	80.29	83.14	86.14	88.0
Glasses (G)	77.57	84.61	84.86	86.86	89.57	90.14	77.57	84.61	84.86	86.86	89.57	90.14	77.14	84.29	84.86	86.71	88.57	90.0
Face mask (FM)	77.57	84.61	84.86	86.86	89.57	90.14	77.57	84.61	84.86	86.86	89.57	90.14	77.14	84.29	84.86	86.71	88.57	90.0
G + M	77.57	84.61	84.86	86.86	89.57	90.14	77.57	84.61	84.86	86.86	89.57	90.14	77.14	84.29	84.86	86.71	88.57	90.0

In the second and third experiments, the recognition rate of RF approach with 22 thermal points is improved significantly. However, in the third experiment, when the parameter N is more than 5, the experimental results for F = 22 are worse than those for F = 12 in the occluded images. The reason why the recognition rate for F = 22 is worse is because the occluded thermal points are used in classifier training. Based on the comparisons between Tables 3 and 4, it is concluded the RF approach with 12 thermal points achieves better performance.

There are some articles targeting issues of thermal image using CNN-based methods [28, 29]. Table 5 lists two experiment results for dynamic feature selection with F = 22 or 12 and CNN approaches. The glasses or mask blocks a large portion of thermal energy, resulting in a loss of information near this region. The system can adaptively choose different feature vector size under different conditions (in normal, noise, glasses, mask, glasses and mask). In the case of occlusion (G, M, and G + M), feature vector size F = 12 has the better performance. On the other hand, in the case of non-occlusion (normal and noise), feature vector size F = 22 has the better performance. In both databases, when the number of training images is less than 10, the results of CNN is worse than those of RF approach. This experiment proves that more samples are required for CNN approach to obtain a better performance. The recognition rate of the proposed RF method is still comparable for the UCH-TTF database. This means the RF approach is competitive while having small database.

**Table 5:** Performance of experiments for dynamic model (RF) with F = 22 or 12 and CNN

	Dynamic model (RF)						CNN					
	N = 5	N = 10	N = 15	N = 20	N = 25	N = 30	N = 5	N = 10	N = 15	N = 20	N = 25	N = 30
PUCV-DTF												
Normal	99.48	100	100	100	100	100	99.13	100	100	100	100	100
Noise	95.87	96.74	97.74	97.83	98.61	99.74	98.26	99.13	99.48	99.74	99.87	99.91
Glasses (G)	98.57	100	100	100	100	100	99.78	100	100	100	100	100
Face mask(FM)	98.57	100	100	100	100	100	99.35	100	100	100	100	100
G + M	98.57	100	100	100	100	100	99.57	100	100	100	100	100
UCH-TTF												
Normal	83.29	84.43	85.71	87.43	89.29	90.29	76.43	84.86	87.86	89.56	94.57	96.70
Noise	79.29	79.29	81.43	82.14	82.86	82.86	75.71	79.28	85.43	88.56	92.29	94.14
Glasses (G)	77.14	84.29	84.86	86.71	88.57	90.0	76.14	84.71	87.29	89.29	93.57	95.99
Face mask(FM)	77.14	84.29	84.86	86.71	88.57	90.0	75.86	83.86	86.99	89.29	93.14	95.70
G + M	77.14	84.29	84.86	86.71	88.57	90.0	74.77	82.14	86.29	88.70	91.14	92.43

**Fig. 7:**
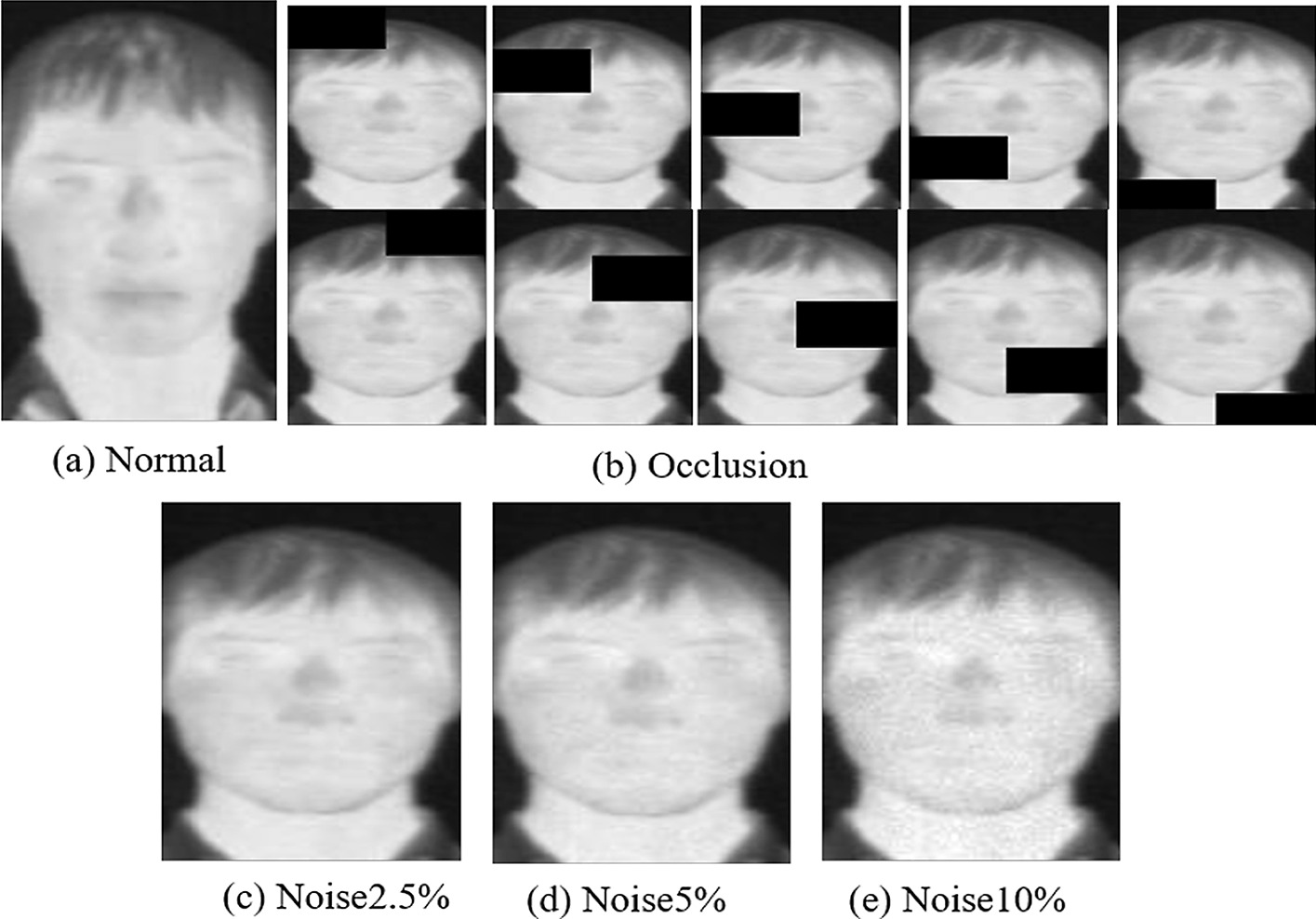
The original and modified experiment samples. **a** Normal images, **b** Occlusion images, **c** Noise2.5% images, **d** Noise5% images, **e** Noise10% images

To demonstrate the robustness of our method, comparisons with 6 methods on UCH-TTF database [17] were conducted as well. As shown in Fig. 7, the original and modified experiment samples were selected from UCH-TTF database. Image is divided into 10 regions for each object (5 rows, 2 columns). One of ten regions is then randomly masked to simulate the situation of occlusion (see Fig. 7b). As shown in Fig. 7c–e, the noise is added to the original coordinates of the image. The percentage of noise is related to the intensity of gray values between the centers of two eyes. The noise levels for each image are randomly selected between 0% and the maximum gray value of 2.5%, 5% or 10% to generate three different levels of noise.

The recognition rates for UCH-TTF together with average (Avg) face recognition and standard deviation (SD) are listed in Table 6. We selected 20 samples from each person of the UCH-TTF dataset as gallery set and the remaining as test sets (normal, occlusion, noise2.5%, noise5% and noise10%). The gallery set contains only normal images without occlusion or noise. The proposed method outperforms other classic appearance based methods, except for the WLD and GJD methods [17]. However, the total feature points per target used in our experiment for training model is 440 (22 feature points with 20 images), which is around one twenty-eighth to one sixtieth of those by other classic methods. Moreover, the standard deviation of the proposed method is 3.32 comparing to 6.8 of GJD and 6.9 of WLD methods. Our proposed method is apparently more robustness.

**Table 6:** Recognition rates for UCH-TTF database

	Normal	Occlusion	Noise2.5%	Noise5%	Noise10%	Avg	SD
Normal	93.10	92.85	96.57	93.71	87.42	92.73	3.32

## Discussion

All experiments are conducted under different conditions. When the facial face is occluded, this results in the difficulty of face recognition. The experiment with occluded images is more difficult than other experiment because the eyes and mouth on face are masked, leading to the loss of important information. In the case of face masking, the performance of recognition is not satisfactory for the RF approach with 22 thermal points. In summary, the dynamic model (RF) with F = 22 or 12 obtains a robust recognition rate under different conditions.

We analyze the relationship between training set and testing set, leading to three different experiments. In Fig. 6 1-a and 2-a, only training the normal images cannot effectively represent the features of different images. As shown in Fig. 6 1-b and 2-b, training set is customized according to the specific image. Therefore, the recognition rate of five experiment images has the best performance, as listed in Tables 3 and 4. This proves better recognition rate comes from more specific training data. In Fig. 6 1-c and 2-c, all experiment images are merged for classifier training to obtain the performance of generalization. Finally, comparing with the PUCV-DTF database, the UCH-TTF database has more temporal variation. This leads to the decrease of the performance.

## Conclusions

In this article, thermal face recognition under different conditions has been proposed. The proposed method can effectively exploit physiological information to perform face recognition. The method has two phases: (1) training phase (2) testing phase. The first phase contains three steps, including preprocessing, feature extraction, and classification. A grid of 22 or 12 thermal points is extracted from the face for generating a feature vector. The feature vector corresponding to each experiment image is exploited for the RF classifier training and the face recognizing in the testing phase. Comparisons with other techniques prove that the proposed method is robust under less feature points, which is around one twenty-eighth to one sixtieth of those by other classic methods. Besides, the standard deviation of the proposed method is one half to one fifth of other methods.

To deal with real world situation, we combine five experiment images (normal, noise, and occlusion) as a training set to improve the robustness and generalization of the model. In comparison with the performance of CNN, the experimental results of the proposed RF method demonstrate its performance against different challenges. The novelty of our method is to use the most representative temperature area on the face for thermal face recognition. Even in occluded situations, experimental results are still stable. On the other hand, it took 69 days for the collection of the UCH-TTF database. This results in the difficulty of thermal face recognition.

## Data Availability

The data that support the findings of this study are available from [17, 18] but restrictions apply to the availability of these data.

## Abbreviations

CNN: convolutional neural network
RF: random forest
IR: infrared
NIR: near infrared
SWIR: short wave infrared
MWIR: medium wave infrared
LWIR: long wave infrared
ROI: region of interest
FLD: Fisher linear discriminant
GMM: Gaussian mixture models
TMP: thermal minuta points
PUCV-DTF: PUCV drunk thermal face
UCH-TTF: UCH thermal temporal face
FC: fully connected layer
